# Tuberculosis knowledge, attitudes, and practices among northern Ethiopian prisoners: Implications for TB control efforts

**DOI:** 10.1371/journal.pone.0174692

**Published:** 2017-03-30

**Authors:** Kelemework Adane, Mark Spigt, Laturnus Johanna, Dorscheidt Noortje, Semaw Ferede Abera, Geert-Jan Dinant

**Affiliations:** 1 Department of Medical Microbiology and Immunology, College of Health Sciences, Mekelle University, Mekelle, Ethiopia; 2 Maastricht University/CAPHRI School for Public Health and Primary Care, Department of Family Medicine, Maastricht, the Netherlands; 3 Maastricht University, Faculty of Health, Medicine and Life Sciences, Maastricht, the Netherlands; 4 Department of Public Health, College of Health Sciences, Mekelle University, Mekelle, Ethiopia; Hospital San Agustin, SPAIN

## Abstract

**Introduction:**

Although awareness is an important component in tuberculosis (TB) control, we do not know how much Ethiopian prisoners know about TB. This study assessed the level of knowledge, attitudes, and practices (KAP) of prisoners about TB in eight northern Ethiopian prisons.

**Methods:**

Data were collected cross-sectionally from 615 prisoners using a standardized questionnaire between March and May 2016. The outcome variables were defined considering the basic elements about TB.

**Results:**

Out of 615 prisoners, only 37.7% mentioned bacteria as a cause of TB while 21.7% related TB to exposure to cold wind. Eighty-eight per cent correctly mentioned the aerial route of TB transmission and 27.3% had perceived stigma towards TB. The majority (63.7%) was not aware of the possibility of getting multi-drug-resistant strains when they would not adhere to treatment. Overall, only 24% knew the basic elements about TB, 41% had favorable attitudes, and 55% had a good practice. Prisoners who were urban residents were generally more knowledgeable than rural residents (adjusted OR = 2.16; 95% CI = 1.15–4.06). Illiterates were found to be less knowledgeable (adjusted OR = 0.17; 95% CI = 0.06–0.46), less likely to have a favorable attitude (adjusted OR = 0.31; 95% CI = 0.15–0.64), and less good practice (adjusted OR = 0.35; 95% CI = 0.18–0.69). Significant differences were also observed between the different study prisons.

**Conclusions:**

Knowledge of prisoners regarding the cause of TB and consequences of non-adherence to TB treatment was low. Knowledge on the transmission, symptoms, and prevention was fairly high. Health education interventions, focused on the cause and the translation of the knowledge to appropriate practices, are needed in all the study prisons. Special attention should be given to less educated prisoners, and to prisons with a high number of prisoners and those in remote areas.

## Introduction

Even though the incidence of tuberculosis (TB) has decreased worldwide, it remains a global health challenge. An estimated 10.4 million people developed TB in the year 2015 of which one-quarter was from Africa [1]. The disease is more prevalent in congregate settings such as prisons [2]. Especially, it is much worse in sub-Saharan prisons due to the added problems of human immunodeficiency virus (HIV) and poverty [3]. In Ethiopian prisons, a four to nine-fold higher prevalence of TB has been reported compared to the general population [4,5].

The global focus of TB control programs is on early diagnosis and treatment of cases in high TB and HIV-endemic areas [1]. However, the low TB case detection rate and the emergence of multi-drug-resistant strains have been a challenge [1,6]. Raising communities' awareness contributes for early diagnosis of TB which is one of the pillars of the End TB Strategy [1]. Studies documented a positive association between TB knowledge and care seeking and treatment adherence [7–9]. However, the level of knowledge should be known, also in relation to previous reports, before informed decisions can be made when designing and implementing appropriate educational interventions. In this regard, studies conducted in the general populations of sub-Saharan countries documented misconceptions ranging from 66.3% to 99.7% of the population on the etiology (cause) of TB, 27.6% to 90.1% on the symptoms, 0.1% to 48.6% on the transmission and 33.4% to 92.9% on prevention methods [10–16]. Stigma towards TB patients has been reported in up to 58.3% of the respondents [10,14]. Literacy status, socio-cultural differences, gender, and spatial variations have been reported to be factors affecting TB knowledge, attitude and practices (KAP) [11,13,16].

Baseline data regarding prisoners' knowledge of TB and related factors are limited. Studies conducted in prisons of Brazil [17] and Texas [18] reported gaps on some specific TB KAP variables. In a Brazilian prison, only 5.0% and 3.6% of the prisoners could mention the TB symptoms and prevention methods, respectively, and in a USA prison 43.0% of the prisoners had a perceived stigma towards TB. To our knowledge, in sub-Saharan prisons, only one study assessing prisoners knowledge was conducted six years ago in Eastern Ethiopian prisons [19]. This study reported a moderate level of knowledge about TB and revealed some misconceptions about its causes, control and prevention. This study was, however, limited in scope in that it did not address the attitude, and was only conducted among presumptive TB cases.

Moreover, in culturally diversified countries like Ethiopia, TB knowledge-level has been reported to show significant spatial variations [16]. In addition, through the Internet and intensive educational campaigns, healthcare information can reach many people quickly and increase the level of knowledge among people [20]. In a previous study among Ethiopian prisons we observed quite some TB cases with long-lasting symptoms without being diagnosed [4], so we expect that KAP among Ethiopian prisoners is still very low. This study aimed at assessing the level of knowledge, attitude, and practices of prisoners about TB and related factors.

## Methods

### Study setting

This study was conducted in eight northern Ethiopian prisons located in the regions Tigray (Mekelle, Abi Adi, Alamata, Humera, and Wukro) and Amhara (Dessie, Debre Tabor, and Finote Selam) between March and May 2016. In 2015, Ethiopia ranked 10th among the 22 high TB burden countries with an estimated TB incidence of 192 per 100,000 people [1]. The country had a registered prison population of 112,361 (136/100,000 persons) in 2010 [21], which is higher than the imprisonment rates observed in some sub-Saharan African countries such as in Kenya (121/100,000 persons), and Malawi (76/100,000 persons) [21].

### Study design and sampling technique

This was a cross-sectional study, which was also part of a baseline measurement for an educational interventional study aimed to increase TB screening and case detection rate in northern Ethiopian prisons. Larger prison centers located in the main cities of Amhara and Tigray regions were considered as eligible while small jails were excluded (n = 22). Larger prisons were defined as institutions that incarcerate people for longer periods of time, such as many years, while small jails were institutions that confine people for shorter periods of time. A multistage cluster sampling technique was employed to randomly select the study prisons and the prisoners. Only prisoners who would stay imprisoned for a year or longer from the date of the selection were included, since this baseline measurement was part of an intervention study. Prisoners younger than 18 years of age and those mentally ill were excluded.

### Sample size determination

The sample size was determined using a single proportion formula, n_**1** =_ z^2^p (1-p)/d^2^, where n_1_ was the initial sample size, considering a confidence level of 95%, an estimated overall proportion of good knowledge about TB of 52% [12], and a precision of 5%. After using a finite population correction, n_2 =_ n_1_/(1+ (n_1_/N)), where N was the total number of the prisoners in the study sites (N = 8,874), multiplying by 1.5 to account for the clustering effect, and adding a 15% non-response rate, we obtained a final sample size of 634. This figure was then proportionally allocated to each prison as per the total numbers of prisoners.

### Questionnaire and interviewing

We used a semi-structured standardized KAP questionnaire to collect data. The questionnaire was designed in English following the WHO guidelines [22] and was translated into the local languages, Amharic and Tigrigna. The questionnaire consisted of 38 questions, divided into two parts. Part one addressed the socio-demographic characteristics and prison history. The second part addressed aspects related to TB knowledge, attitude, and practices. Briefly, questions regarding the etiology, transmission, prevention, and treatment of TB, beliefs, and feelings about TB and TB patients were included. The interviewing was done by trained data collectors (prison nurses, or trained inmates). For sites with a shortage of prison health professionals, prisoners who were relatively educated (some with a diploma in clinical nursing) were recruited and trained for two days on how to undertake the interview. The interviewing process was closely monitored by the investigators in which the first one-fourth of the interviews were monitored by listening and observing the interviewing process. The investigators also stayed close around the interviewing area to assist the interviewers on call for any ambiguity for the rest of the interviews.

### Statistical analysis

Data were entered in EpiData version 3.1 software and the analysis was performed using SPSS version 20.0. Descriptive statistics was used to report frequencies and proportions. Bivariate and multivariate logistic regression analysis was performed to examine the association of independent variables with our outcome variables. Our outcome variables of interest were knowledge about TB, attitude towards TB, and practice. We checked whether there was a clustering effect at the prison level for the outcome variables following the mixed procedure for a possible consideration of the multilevel logistic model. We found, however, that there was no statistically significant variability in the intercepts of the outcome variables across the prison sites; the p values for the intercept estimates of knowledge, attitude, and practice were 0.14, 0.25, and 0.13, respectively.

Knowledge was assessed considering the following crucial elements: able to recognize germ/bacteria as a cause of TB, able to recognize the airborne route of transmission, able to recognize a cough of 2 weeks and longer as a symptom, able to realize covering mouth and nose when coughing/sneezing as a prevention measure, and able to know the free TB treatment availability. Prisoners that mentioned all these five items were categorized as having a ‘good’ knowledge and those who missed one or more of these items were categorized as having ‘poor’ knowledge. Attitude was assessed using three questions: able to mention that TB is a very serious disease, showing a favorable reaction if suspected having TB related symptoms (i.e. seeking health care instead of being ashamed of or hopeless), and showing a compassion and desire to help people with TB. Prisoners that mentioned these three items were categorized as having a ‘favorable’ attitude and the others were categorized as having ‘unfavorable’ attitude. Similarly, practice was assessed using two questions: preference of modern health care for treatment and the intention to visit the facility as soon as realizing having a TB related symptom. Prisoners who mentioned these two items were categorized as having a 'good’ practice and the rest were categorized as having a ‘poor’ practice. All the potential predictor variables were tested against the dichotomized knowledge, attitude, and practice. Multi-collinearity among the independent variables was assessed considering the variance inflation factor of greater than 10 (for our data, the maximum was 3.86). Covariates with p-values of ≤ 0.25 in the bivariate analysis were considered for inclusion in the multivariate model. Accordingly, the multivariate models for the level of knowledge, attitude, and practice consisted of five, four, and six variables, respectively. Educational status and prison site were included in the three models. In addition, age group and occupation were added to the final models of the knowledge and practice level whereas residence was included in the knowledge and attitude model. Duration of imprisonment was added to the attitude model and knowledge and attitude level to the practice model. Comparisons between subgroups with the outcomes were expressed as odds ratios (OR) with a 95% confidence interval (CI). A p-value of ≤ 0.05 was considered to declare a statistically significant association.

### Ethical consideration

The study protocol was approved by the ethical review committee of the College of Health Sciences, Mekelle University. All participants were asked for a written informed consent, and those consented were enrolled. For all illiterate participants, data collectors informed each of them and confirmed the willingness of the participants to sign the informed consent sheet. The consent procedure for these illiterate participants was also approved by the ethics review committee.

## Results

### Socio-demographic characteristics

Of the total number of invited participants (n = 634), five (1%) refused to participate in the study. Fourteen questionnaires with missing demographic characteristics were excluded, so the final analysis was performed with 615 participants. The number of participants included ranged from 22 in Abi Adi to 173 in Mekelle prison. Of the 615 participants, the majority 597 (97%) was male. The median age was 28 years with an interquartile range (IQR) of 12 years. The median duration of imprisonment was 13 months (IQR = 29 months). Almost half (46%) of the participants was farmer and 13% was illiterate. Thirty-seven participants (6%) reported a history of having TB disease.

### Knowledge about tuberculosis

The prisoners' knowledge about TB is shown in Table 1. In this study, only 37.7% of the participants recognized germ/ bacteria as a cause of TB. Twenty-two percent mentioned that it is mainly caused by exposure to cold. The majority (88.0%) correctly mentioned that TB is transmitted through coughing droplets and 65.7% mentioned covering the mouth when coughing/sneezing as a measure to prevent TB. However, 11.9% and 15.0% of the participants mentioned inappropriate methods including keeping windows closed when they are with chronically coughing people and/or TB patients in a room and avoiding shaking hands, respectively. The majority (88.3%) described that TB is curable with modern drugs, but about one-third (35.5%) did not know the free TB treatment availability.

**Table 1:** Knowledge of northern Ethiopian prisoners about tuberculosis stratified by the status of the previous history of TB disease, 2016 (N = 615).

Variable	Had history of TB, n (%)	Had no TB ever, n (%)	Total, n (%)	p-value^**^
	n = 37	n = 578	N = 615	
**Cause of TB**				0.65
Bacteria*	17 (45.9)	215 (37.2)	232 (37.7)	
Cold wind	5 (13.5)	128 (22.1)	133 (21.7)	
Smoking	3 (8.2)	43 (7.4)	46 (7.5)	
Spoiled soil (soil with a bad odor)	5 (13.5)	61 (10.6)	66 (10.7)	
Poor hygiene	2 (5.4)	53 (9.2)	55 (8.9)	
Don't know	5 (13.5)	60 (10.4)	65 (10.6)	
Others^¶^	0 (0)	18 (3.1)	18 (2.9)	
**Mode of transmission**				0.45
Through coughing droplets*	36 (97.3)	506 (87.6)	542 (88.0)	
Through shaking hands	0 (0)	28 (4.8)	28 (4.6)	
Though sharing dish	0 (0)	22 (3.8)	17 (2.8)	
Don't know	1 (2.7)	22 (3.8)	28 (4.6)	
**Signs and symptoms**^**+**^				
Cough for 2 weeks or above*	28 (75.7)	458 (79.2)	486 (79.1)	0.67
Hemoptysis	20 (54.1)	278 (48.1)	298 (48.5)	0.51
Weight loss	16 (43.2)	272 (47.1)	288 (46.8)	0.73
Ongoing fatigue	11 (29.1)	190 (32.9)	201 (32.7)	0.85
Persistent fever	12 (42.4)	157 (27.2)	169 (27.5)	0.57
Don't know	1 (2.7)	35 (6.1)	36 (5.9)	0.72
Others^¶¶^	40 (108.1)	347 (60.1)	387 (62.9)	1.00
**Prevention methods**^**+**^				
Cover mouth when coughing/sneezing*	23 (62.2)	381 (65.9)	404 (65.7)	0.72
Washing hands	11 (29.7)	188 (32.5)	199 (32.4)	0.86
Avoiding handshakes	6 (16.2)	86 (14.9)	92 (15.0)	0.81
Isolating TB patients	12 (32.4)	214 (37.1)	226 (36.7)	0.73
Closing windows	4 (10.8)	69 (11.9)	73 (11.9)	1.00
Avoid sharing dishes	10 (27.0)	163 (28.2)	173 (28.1)	1.00
Vaccination	10 (27.0)	169 (29.2)	179 (29.1)	0.85
Good nutrition	14 (37.8)	130 (22.5)	144 (23.4)	0.04
Don’t know	0 (0)	27 (4.7)	27 (4.4)	0.39
**Is TB curable**				0.16
Yes	36 (97.3)	507 (87.7)	543 (88.3)	
No	1 (2.7)	71 (12.3)	72 (11.7)	
**Know free TB treatment availability**				0.59
Yes*	26 (70.1)	371 (64.2)	397 (64.5)	
No	11 (29.9)	207 (35.8)	218 (35.5)	
**Risk of defaulting from treatment**^**+**^				
Death	28 (75.7)	458 (79.2)	486 (79.1)	0.54
Relapse	12 (32.4)	256 (44.3)	268 (43.6)	0.17
No cure	13 (35.1)	232 (40.1)	245 (39.8)	0.61
Drug resistance	17 (45.9)	206 (35.6)	223 (36.3)	0.22
Don’t know	2 (5.4)	8 (1.4)	10 (1.6)	1.00
**Vulnerability for TB**				0.43
Prisoners	26 (70.3)	440 (76.2)	466 (75.8)	
General community	11 (29.7)	138 (23.8)	149 (24.2)	

*: the five crucial elements

¶: curse/demon, malnutrition

**+:** multiple responses possible

¶¶: chest pain, skin rash, nausea, severe headache

**: p-value from the chi-square test

### TB knowledge and associated factors

Table 2 shows the relationships between TB knowledge, using the five crucial points, and potential predictor variables. Of 615 participants, 149 (24%; 95% CI = 21%-28%) had a good level of knowledge about TB. Government employees had a significantly higher level of knowledge compared to farmers (AOR 2.92; 95% CI = 1.21–7.03). Prisoners that were urban dwellers were more knowledgeable than rural prisoners (AOR = 2.16; 95% CI = 1.15–4.06). There was also an association between the level of education and TB knowledge. Another interesting finding was that there appeared to be a significant variation in the level of TB knowledge across the study prisons: compared to prisoners of Mekelle, prisoners of Debre Tabor (AOR = 2.71; 95% CI = 1.36–5.42), and Finote Selam (AOR = 4.16; 95% CI = 2.05–8.43) were more knowledgeable, whereas those imprisoned in Wukro (AOR = 0.12; 95% CI = 0.03–0.57), and Humera (AOR = 0.21; 95% CI = 0.06–0.74) were less knowledgeable.

**Table 2:** Factors related to the level of knowledge about tuberculosis among northern Ethiopian prisoners in the bivariate and multivariate logistic regression analysis (N = 615).

Variable	Knowledge level			
	Good, n (%)	Poor, n (%)	COR (95% CI)	AOR (95% CI)
**All participants (N = 615)**	149 (24)	466 (76)		
**Age, years**				
18–45	136 (25)	406 (75)	1.55 (0.82–2.91)	1.08 (0.51–2.26)
≥ 46	13 (18)	60 (82)	ref	
**Educational status**				
Illiterate	7 (9)	72 (91)	0.13 (0.06–0.29)	0.17 (0.06–0.46)
Read and write only	10 (14)	62 (86)	0.22 (0.1–0.43)	0.21 (0.08–0.52)
Primary	42 (16)	215 (84)	0.25 (0.16–0.39)	0.27 (0.15–0.48)
Secondary or above	90 (44)	117 (56)	ref	
**Occupation before imprisonment**				
Government employee	45 (60)	30 (40)	8.11 (4.62–14.3)	2.92 (1.21–7.03)
Student	22 (24)	69 (76)	1.73 (0.97–3.07)	0.77 (0.33–1.76)
Unemployed	5 (3)	12 (97)	2.25 (0.76–6.72)	0.53 (0.13–2.09)
Private worker	33 (22)	117 (78)	1.53 (0.92–2.52)	0.59 (0.26–1.33)
Farmer	44 (16)	238 (84)	ref	
**Residence**				
Urban	95 (34)	185 (66)	2.67 (1.82–3.92)	2.16 (1.15–4.06)
Rural	54 (16)	281 (84)	ref	
**History of TB**				
Yes	7 (19)	30 (91)	0.72 (0.31–1.67)	-
No	142 (25)	436 (75)	ref	
**Length of imprisonment**				
>12 months	74 (19)	323 (81)	0.92 (0.64–1.33)	-
≤12 months	75 (34)	143 (66)	ref	
**Prison site**				
Wukro	2 (5)	40 (95)	0.15 (0.04–0.65)	0.12 (0.03–0.57)
Abi-Adi	4 (18)	18 (92)	0.67 (0.22–2.09)	1.23 (0.37–4.47)
Humera	3 (5)	57 (95)	0.16 (0.05–0.53)	0.21 (0.06–0.74)
Alamata	11 (23)	37 (77)	0.89 (0.42–1.92)	1.58 (0.66–3.79)
Dessie	28 (27)	75 (73)	1.13 (0.65–1.96)	1.12 (0.58–2.14)
Debre Tabor	27 (34)	53 (66)	1.54 (0.86–2.74)	2.71 (1.36–5.42)
Finote Selam	31 (36)	56 (64)	1.67 (0.96–2.92)	4.16 (2.05–8.43)
Mekelle	43 (25)	130 (75)	ref	

AOR: adjusted odds ratio; COR: crude odds ratio; CI: confidence interval; TB: tuberculosis; ref: reference

### Attitudes and practices about tuberculosis

The majority of the participants (84.2%) believed that TB is a very serious disease. Sixty-nine percent mentioned that they would not feel feared or ashamed when they would have TB symptoms, but would simply visit a health care facility. A considerable proportion (27.3%) had stigmatizing thoughts towards TB patients (i.e. they mentioned that they fear such people and would stay away from them or would not show any feeling instead of showing compassion and a desire to help). The majority (82.6%) preferred to visit modern health care (Table 3).

**Table 3:** Attitudes and practices of northern Ethiopian prisoners about tuberculosis stratified by the status of the previous history of TB disease, 2016 (N = 615).

Variable	Had history of TB, n (%)	Had no TB ever, n (%)	Total, n (%)	p-value^**^
	n = 37	n = 578	N = 615	
**Thought on seriousness of TB diseases**				0.23
Very serious*	30 (81.1)	488 (84.4)	518 (84.2)	
Somewhat serious	4 (10.8)	71 (12.3)	75 (12.2)	
Not serious	3 (8.1)	19 (3.3)	22 (5.6)	
**Reaction if had TB symptoms**				0.29
Fear	6 (16.2)	115 (19.9)	121 (19.7)	
Shame	2 (5.4)	23 (4.0)	25 (4.1)	
Sadness/hopelessness	4 (18.8)	40 (6.9)	44 (7.2)	
Visit health facility*	25 (67.6)	400 (69.2)	425 (69.0)	
**Feelings about people with TB diseases**				0.69
I feel compassion and a desire to help*	30 (81.1)	417 (72.1)	447 (72.7)	
I feel compassion, but stay away from such people	4 (10.8)	111 (19.2)	115 (18.7)	
I fear them because they may infect me	2 (5.4)	35 (6.1)	37 (6.0)	
I have no particular feeling	1 (2.7)	15 (2.6)	16 (2.6)	
**Choice for TB treatment**				0.027
Modern health care^¶^	29 (78.4)	479 (82.9)	508 (82.6)	
Traditional healers	5 (13.5)	23 (4.0)	28 (4.5)	
Holy water	1(2.7)	39 (6.7)	40 (6.6)	
Don’t know	2 (5.4)	37 (6.4)	39 (6.3)	
**Time point to visit health facility**				0.73
When treatment of my own does not work	0 (0)	16 (2.8)	16 (2.6)	
As soon as realizing the symptoms might be related to TB^¶^	24 (64.9)	376 (65.1)	400 (65.0)	
After 3–4 weeks of having symptoms	12 (32.4)	165 (28.5)	177 (28.8)	
I would not go to a doctor	1 (2.7)	21 (3.6)	22 (3.6)	

TB: Tuberculosis

*: the three crucial elements for attitude

¶: the two crucial elements for practice

**: p-value from the chi-square test

### Overall attitude and associated factors

The attitude of prisoners towards TB and the relationship with the potential predictor variables is summarized in Table 4. Less than a half (41%; 95% CI = 37–45) of the prisoners had a favorable attitude towards TB. Respondents that were able to mention that TB is a very serious disease, those showing a favorable reaction if suspected having TB related symptoms and a desire to help people with TB were considered as having a favorable attitude. Significant variations in the level of attitude were observed by the educational level and study sites. By occupation, students (AOR = 1.88; 95% CI = 1.1–3.5) were found to have a higher level of favorable attitude compared to farmers.

**Table 4:** Factors related to the level of attitude about tuberculosis among northern Ethiopian prisoners in the bivariate and multivariate logistic regression analysis (N = 615).

	Level of attitude			
Variable	Favorable, n (%)	Unfavorable, n (%)	COR (95% CI)	AOR (95% CI)
**All participants (N = 615)**	250 (41)	365 (59)		
**Age, years**				
18–45	220 (41)	322 (59)	0.98 (0.59–1.61)	-
≥46	30 (4)	43 (96)	ref	
**Educational status**				
Illiterate	19 (24)	60 (76)	0.25 (0.14–0.45)	0.31 (0.15–0.64)
Read and write only	20 (3)	52 (97)	0.31 (0.17–0.55)	0.34 (0.16–0.68)
Primary	96 (37)	161 (63)	0.48 (0.33–0.69)	0.46 (0.29–0.72)
Secondary or above	115 (56)	92 (44)	ref	
**Occupation before imprisonment**				
Government employee	33 (44)	42 (56)	1.88 (1.12–3.18)	0.92 (0.44–1.94)
Student	50 (55)	41 (45)	2.92 (1.79–4.75)	1.88 (1.1–3.5)
Unemployed	8 (47)	9 (53)	2.13 (0.79–5.74)	1.38 (0.44–4.41)
Private worker	76 (51)	74 (49)	2.46 (1.63–3.71)	1.41 (0.76–2.62)
Farmer	83 (29)	199 (71)	ref	
**Residence**				
Urban	138 (49)	142 (51)	1.94 (1.39–2.68)	0.93 (0.56–1.53)
Rural	112 (33)	223 (67)	ref	
**History of TB**				
Yes	14 (38)	23 (62)	0.88 (0.44–1.75)	-
No	236 (41)	342 (59)	ref	
**Length of imprisonment**				
>12 months	161 (41)	236 (59)	1.12 (0.81–1.55)	-
≤12 months	89 (40)	129 (60)	ref	
**Prison site**				
Wukro	9 (21)	33 (79)	0.3 (0.14–0.67)	0.29 (0.13–0.68)
Abi Adi	14 (64)	8 (36)	1.94 (0.78–4.87)	2.44 (0.93–6.39)
Humera	30 (50)	30 (50)	1.11 (0.61–1.99)	1.42 (0.74–2.71)
Alamata	2 (4)	46 (96)	0.05 (0.01–0.21)	0.06 (0.01–0.24)
Dessie	57 (53)	46 (47)	1.38 (0.84–2.24)	1.42 (0.75–2.71)
Debre tabor	24 (30)	56 (70)	0.48 (0.27–0.83)	0.56 (0.31–1.03)
Finote selam	32 (37)	55 (63)	0.65 (0.38–1.09)	0.94 (0.53–1.68)
Mekelle	82 (47)	91 (53)	ref	

AOR: adjusted odds ratio; COR: crude odds ratio; CI: confidence interval; TB: tuberculosis; ref: reference

### Overall practice and associated factors

More than half of the prisoners, 55% (95% CI = 51.1–58.9), had a good practice related to TB. There was a significant variation in practice by the level of education and study sites. Having a good TB knowledge (COR = 1.49; 95% CI = 1.03–2.18) and a favorable attitude (COR = 1.41; 95% CI = 1.02–1.95) were also associated with having a good practice but was only in the bivariate analysis (Table 5).

**Table 5:** Factors associated with the level of practice towards tuberculosis among northern Ethiopian prisoners in the bivariate and multivariate logistic regression analysis (N = 615).

	Level of practice			
Variable	Good, n (%)	Poor, n (%)	COR (95% CI)	AOR (95% CI)
**All participants(N = 615)**	338 (55)	277 (45)		
**Age, years**				
18–45	293 (54)	249 (46)	0.73 (0.44–1.01)	0.74 (0.42–1.29)
≥46	45 (62)	28 (38)	ref	
**Educational status**				
Illiterate	31 (39)	48 (61)	0.41 (0.24–0.69)	0.35 (0.18–0.69)
Read and write only	40 (56)	32 (44)	0.79 (0.46–1.36)	0.55 (0.27–1.11)
Primary	140 (55)	117 (45)	0.75 (0.52–1.09)	0.7 (0.44–1.11)
Secondary or above	127 (61)	80 (39)	ref	
**Occupation before imprisonment**				
Government employee	48 (64)	27 (26)	1.52 (0.89–2.57)	0.94 (0.48–1.85)
Student	48 (53)	43 (47)	0.96 (0.59–1.53)	0.65 (0.36–1.15)
Unemployed	8 (47)	9 (53)	0.76 (0.29–2.21)	0.51 (0.18–1.45)
Private worker	82 (55)	68 (45)	1.03 (0.69–1.54)	0.82 (0.49–1.38)
Farmer	152 (54)	130 (46)	ref	
**Residence**				
Urban	159 (57)	121 (43)	1.15 (0.83–1.58)	-
Rural	179 (53)	156 (47)	ref	
**History of TB**				
Yes	21 (57)	16 (43)	1.08 (0.55–2.11)	-
No	317 (55)	261 (45)	ref	
**Length of imprisonment**				
>12 months	203 (51)	194 (49)	1.05 (0.76–1.44)	-
≤12 months	135 (62)	83 (38)	ref	
**Prison site**				
Wukro	22 (52)	20 (48)	0.8 (0.41–1.58)	0.95 (0.47–1.94)
Abi-Adi	13 (59)	9 (41)	1.05 (0.43–2.59)	1.18 (0.46–3.1)
Humera	20 (25)	40 (75)	0.37 (0.19–0.68)	0.38 (0.2–0.72)
Alamata	15 (32)	33 (68)	0.33 (0.17–0.66)	0.39 (0.19–0.82)
Dessie	53 (51)	50 (49)	0.77 (0.47–1.26)	0.77 (0.46–1.23)
Debre Tabor	58 (72)	22 (28)	1.93 (1.08–3.42)	2.18 (1.19–4.0)
Finote Selam	57 (66)	30 (34)	1.39 (0.81–2.36)	1.44 (0.8–2.58)
Mekelle	100 (58)	73 (42)	ref	
**Knowledge**				
Good	93 (62)	56 (38)	1.49 (1.03–2.18)	1.05 (0.67–1.63)
Poor	245 (53)	221 (47)	ref	
**Attitude**				
Favorable	150 (60)	100 (40)	1.41 (1.02–1.95)	1.32 (0.92–1.92)
Unfavorable	188 (52)	177 (48)	ref	

AOR: adjusted odds ratio; COR: crude odds ratio; CI: confidence interval; TB: tuberculosis; ref: reference

## Discussion

This study revealed gaps in knowledge, attitudes and practices among northern Ethiopian prisoners with regard to TB. Only about four out of ten prisoners were able to recognize germ/bacteria as a cause of TB. The large majority related TB to exposure to cold wind, spoiled soil (a soil with a bad odor), poor hygiene, and smoking. The majority (63.7%) was not aware of the possibility of getting multi-drug-resistant strains due to treatment non-adherence. Overall, only 24% knew the basic elements about TB, 41% had favorable attitudes, and a bit more than a half (55%) had a good practice towards TB. TB knowledge, level of attitude and practice were all significantly related to the level of education, and study sites.

When compared to a previous report from Eastern Ethiopian prisons, the proportion of prisoners that mentioned the correct cause of TB is remarkably high in our study (37.7% vs. 1.6%) [19]. Knowledge on the transmission (88.0% vs. 74.1%) and free treatment availability (64.5% vs. 50.3%) is also higher in the current study. This discrepancy could be due to the time difference; knowledge level could vary over time owing to the routine health education activities and improved public media access [23]. The prison health personnel reported that they sometimes provide health education to prisoners about health issues in general. The spatial difference, and associated socio-cultural differences, between the two Ethiopian studies might also partly explain the discrepancy [16]. Similarly, the knowledge about the cause of TB (37.7%), a cough of 2 weeks or more as a TB symptom (79.1%), the airborne route of transmission (88.0%), and covering mouth/nose when coughing as a method of prevention (65.7%) was also higher when compared with reports from the general population in different parts of Ethiopia [10–14,24]. One explanation of this variation could be the fact that prisons are ideal settings for health interventions [25]; in prisons, it is easy to provide health education since a lot of people can be reached at one time compared to the scattered remote areas of the general population.

Very specific comparison with prisons of other sub-Saharan countries was not possible due to the lack of similar published data. However, studies conducted in Brazilian [17] and USA (Texas) [18] prisons reported a much lower knowledge level. In addition to the time difference, the difference in the prevalence of TB might explain the discrepancy, especially for the prison in the USA [26]. In those areas, prisoners might not give much attention to TB while it is a common issue in high burden areas.

In general, from the above comparisons, we see that northern Ethiopian prisoners had a fairly high level of knowledge about the symptoms, transmission, and prevention of TB. This might suggest good health seeking behavior among our prisoners since several studies showed an association between knowledge of TB and early treatment seeking [7,9]. However, this seems not entirely true in daily practice since in our previous TB survey, half of the TB cases were left undiagnosed for long periods; some were even coughing for more than two years [4]. Other factors such as a poor referral system might also contribute significantly to severe treatment delay [4]. Therefore, we suggest that educational interventions in the study prisons should not only focus on delivering the message, but should also ensure that the knowledge gained can lead to appropriate treatment through regular monitoring and evaluation and clearly defined treatment protocols. We also observed a relatively high level of knowledge on the cause of TB; but, this can be called not good enough as the majority (62%) remained having several misconceptions. The ‘exposure to cold wind’ was the most frequently mentioned misconception. This is not surprising as this misconception is common in Ethiopia as shown by previous studies from the general population [11–13], and eastern Ethiopian prisons six years ago [19]. This misconception could still be an important catalyst for the spread of TB, especially in the severely overcrowded Ethiopian prisons [4,27]. It is completely contra-effective if people would tend to keep their windows closed to protect them from the "cold wind", while ventilation is important in preventing TB. Our observation substantiates this report in that 12% mentioned keeping windows closed as a preventive measure when they are with chronically coughing people and/or TB patients in a room. This issue should be stressed when delivering health education in Ethiopian prisons.

In the current study, about one-fourth had perceived stigma towards TB patients which is slightly higher than the study from Brazil (21.6%) [17], but lower than those observed in the USA (43.0%) [18], Amhara region (58.3%) [10], and southwest Ethiopia (51.2%) [14]. The relatively favorable outcome on this variable in our study could be the result of routine health education that has been delivered to the prisoners. In our study prisons, the prison health professionals reported that they provide routine health education to prisoners on infectious diseases including TB. Stigmization has been shown to have a direct impact on health care seeking and infection prevention [7]. In order to avoid the social isolation, TB patients may deliberately conceal their status, infecting many more people. This would particularly have severe consequences in prison settings. While the relatively low level of stigmatization in our study is encouraging, further efforts are still needed to ensure more acceptable and useful attitudes towards TB.

With regard to the practice, although the majority (82.6%) had preference to the modern health care, it is worthy to note that a substantial proportion preferred traditional healers (herbs) and holy water sites as a first priority for treatment. This is in line with the previous studies from eastern Ethiopian prisons [19] and reports from the general population in Tigray [12], Amhara [10], and Afar [11] regions. Such misconceptions should be targeted as they contribute to treatment delay and spreading of the disease [7,8]. However, TB control in sub-Saharan prisons remains neglected by policy makers [28]. Studies suggest the need to shift from considering TB in prisons as a problem of the prison population to considering it as a problem to the larger civilian community [29,30]. We suggest that, for the national TB program to be successful, the concerned bodies such as the federal ministry of health, regional health bureaus, and non-governmental organizations (NGOs) should give priority attention to Ethiopian prisons and use this scientific evidence to help design and provide appropriate health education in the settings. The prison health personnel should be given a sensitization training tailored at the identified misconceptions and continuous support is needed to ensure the quality and the sustainability of the health education.

We also identified some factors that were associated with prisoner's KAP of TB. The level of education was one of the independent predictors for KAP which is consistent with previous reports in Ethiopia [12,13] and elsewhere [31,32]. This relationship could be taken as an opportunity to consider peer mentorship; relatively educated prisoners could be trained and provide routine health education to their fellow inmates, especially in resource-limited prisons of the sub-Saharan Africa. By occupation, prisoners who were government employs were more likely to have a higher awareness about TB. This is an expected relation as these groups are highly educated compared to farmers. There was also knowledge variability by residence; urban dwellers being more knowledgeable. This is in agreement with previous findings [12]. Differences in literacy status, and access to health service and media might justify the relation.

There was also a significant variation of the TB KAP across the study sites. Compared to prisoners of Mekelle, prisoners of Debre Tabor and Finote Selam were more knowledgeable, whereas those imprisoned in Wukro and Humera were less knowledgeable. This local variation is consistent with the previous reports from Eastern Ethiopian prisons [19] and Ghana [16]. From our observations, the number of health personnel (prison nurses) and the availability of a media center in the study prisons were roughly comparable. Mekelle prison had a slightly better medical clinic and more adequate drug stocks. The differences in the commitment of the prison health personnel in delivering health information might otherwise justify the discrepancy. Humera prison is in a remote area with a relatively harsh weather condition which might influence health personnel's routine activities. These findings might indicate the need to consider geographical differences when designing and implementing educational interventions on TB so as to make the maximum impact.

Some limitations of the study should be mentioned. The fact that we used different interviewers for different prisons might have influenced the consistency of the interview. Our study also shares limitations of other studies that use KAP questionnaires. The truthfulness of the respondents' answers remains difficult, especially for the attitude and belief items [17]. Considering these issues, the data collectors were well trained and informed not to give leading questions and strict supervision was done throughout the interview process.

## Conclusions

This study revealed that knowledge of prisoners about the cause of TB and consequences of non-adherence to TB treatment was low. Knowledge on the transmission, symptoms, and prevention was fairly high. However, the overall knowledge on the basic elements of TB remains low and appeared to significantly vary by study site, and socio-demographic characteristics of prisoners. Health education interventions, focused on the cause and the importance of early diagnosis and treatment are needed in all the study prisons, but special attention should be given to less educated prisoners, and prisons with a high number of inmates and those in remote areas.

## Supporting information

S1 File (SAV)

S2 File (PDF)
