# Supplementary material for: Tuberculosis knowledge, attitudes, and practices among northern Ethiopian prisoners: Implications for TB control efforts
Source: PLoS One. 2017 Mar 30;12(3):e0174692. doi: 10.1371/journal.pone.0174692 (PMC5373603; doi:10.1371/journal.pone.0174692)
Supplement: S2 File — (PDF) [file pone.0174692.s002.pdf]

Date __________ Name of the prison____________
Code_____________Room No.____________Block No.____________
Name of interviewer_________________ Mobile No__________________

**KAP Survey questionnaire for Ethiopian prisoners**		
**Part I: Socio-demographic characteristics**		
S.No	Questions	Coding category/response
1.	Age in years	______________________ years
2.	Sex	1. Female<br>2. Male
3.	Marital status	1. Single<br>2. Married<br>3. Divorced<br>4. Widowed
4.	Education	1. Not able to read and write<br>2. Able to read and write without formal school year<br>3. Primary (1-8)<br>4. Secondary (9-10 or 12)<br>5. College (10+ or 12+)<br>6. University degree
5.	Occupation before imprisonment	1. Civil servant(gov't)<br>2. Farmers<br>3. Private worker<br>4. Student<br>5. Unemployed<br>6. Others (specify___________________)
6.	Residence place (before imprisonment)	1. Rural<br>2. Urban
7.	Type of prisoner	1. New entrant<br>2. Not new entrant but on process<br>3. Not new entrant and sentenced
8.	How long have you imprisoned in this prison?	______________________months
9.	Have you ever been had a TB disease?	1. Yes<br>2. No
	**Part II: TB knowledge and awareness**	
10.	What do you think are causes of TB? (chose only one)	1. Bad luck/curse<br>2. Bacteria (germs)<br>3. Demon<br>4. Cold wind<br>5. Spoiled soil (soil with a bad odor)

Date __________ Name of the prison____________
Code_____________Room No.____________Block No.____________
Name of interviewer_________________ Mobile No__________________

		6. Smoking<br>7. Poor hygiene<br>8. Malnutrition<br>9. I don't know<br>10. Others (specify) ……………..
11.	Do you know how can a person get infected with TB?(chose only one)	1. Through handshake<br>2. Through the air when the infected person coughs or sneezes<br>3. Through sharing food with infected person<br>4. By clothes with infected person<br>5. Don't know<br>6. Other (specify) -------------------
12.	What are the signs and symptoms of TB? (Please check all that are mentioned)	1. Rash<br>2. Cough that lasts longer than 2 weeks<br>3. Coughing up blood<br>4. Severe headache<br>5. Nausea<br>6. Weight loss<br>7. Fever without clear cause that lasts more than 7 days<br>8. Chest pain<br>9. Shortness of breath<br>10. Ongoing fatigue<br>11. Do not know<br>12. Others (specify) _________________________
13.	How can we prevent our self from being infected by TB? (Please check all that are mentioned.)	1. Avoid shaking hands<br>2. Covering mouth and nose when coughing or sneezing<br>3. Avoid sharing dishes<br>4. Washing hands after touching items in public places<br>5. Closing windows at home<br>6. Through good nutrition<br>7. By praying<br>8. By vaccination<br>9. By isolating TB patients<br>10. Do not know<br>11. Others (specify)

Date __________ Name of the prison____________
Code_____________Room No.____________Block No.____________
Name of interviewer_________________ Mobile No__________________

14.	What is the best treatment for TB? (chose only one)	1. Herbal Remedies<br>2. Home Remedies<br>3. Praying /holy water<br>4. Don't Know<br>5. Modern drugs
15.	According to you, can a TB affected person be cured?(chose only one)	1. Yes, completely<br>2. Yes, partially<br>3. No
16.	If yes, how long should the treatment be taken to cure TB?	__________ weeks/months or don't know
17.	Do you know any danger if a TB patient is not taking treatment? (Please check all that are mentioned)	1. Death<br>2. Infects others<br>3. Losses weight<br>4. Develops sever health problems<br>5. I don't know<br>6. Others (specify) _________________
18.	Do you know any danger if a TB patient is not taking the drug properly or defaulted? Please check all that are mentioned.)	1. Death<br>2. Relapse<br>3. Inability to cure infection<br>4. Drug resistance<br>5. Don't know
**B. Attitude and practices about TB**		
19.	In your opinion, how serious disease is TB? (chose only one)	1. Very serious<br>2. Somewhat serious<br>3. Not very serious
20.	Do you afraid to get infected with TB? (chose only one)	1. Yes<br>2. No
21.	What would be your reaction if you were found out that you have TB? (chose only one)	1. Fear<br>2. Surprise<br>3. Shame<br>4. Sadness or hopelessness<br>5. Seek immediate medication
22.	What would you do if you thought you had symptoms of TB? (chose only one)	1. Go to a health facility<br>2. Go to pharmacy<br>3. Got to traditional healer<br>4. Pursue other self-treatment options (herbs, etc.)<br>5. Others (specify) __________________

Date __________ Name of the prison____________
Code_____________Room No.____________Block No.____________
Name of interviewer_________________ Mobile No__________________

23.	If you had symptoms of TB, at what point would you go to the health facility? (chose only one)	1. When treatment on my own does not work.<br>2. When symptoms that look like TB signs last for 3–4 weeks.<br>3. As soon as I realize that my symptoms might be related to TB.<br>4. I would not go to the doctor.
24.	If you would not go to the health facility, what is the reason? (Please check all that apply)	1. Not sure where to go<br>2. Cost<br>3. Difficulties with transportation/distance to clinic<br>4. Do not trust medical workers<br>5. Do not like attitude of medical workers<br>6. Prison personnel reuses to refer me<br>7. Other (specify) ___________________
25.	How expensive do you think TB treatment is in Ethiopia? (chose only one)	1. It is free of charge<br>2. It is reasonably priced<br>3. It is somewhat/moderately expensive<br>4. It is very expensive
26.	Which do you think? Is TB higher in prisons or in the community? (chose only one)	1. In prisons<br>2. In the community
27.	If you say in prisons, what do you think are the factors for this high risk? (Please check all that apply)	1. Inadequate access to food and health services<br>2. Overcrowding<br>3. Poor ventilation<br>4. Easily acquiring TB infection<br>5. Don't know<br>6. Others (specify) __________________
28.	Which statement is closest to your feeling about people with TB disease?	1. "I feel compassion and desire to help."<br>2. "I feel compassion, but I tend to stay away from these people."<br>3. "It is their problem and I cannot get TB."<br>4. "I fear them because they may infect me."<br>5. "I have no particular feeling."<br>6. Other (please explain): _______________

Thank you!
